# Surgical Anatomy of the Supraretinacular Fat Pad: Sensory Innervation and Preservation in Open Carpal Tunnel Release

**DOI:** 10.1227/ons.0000000000001367

**Published:** 2024-09-23

**Authors:** Ildefonso Muñoz Romero, Robbert G. E. Notenboom, Martijn J. A. Malessy

**Affiliations:** ‡ Department of Neurosurgery, Leiden University Medical Center, Leiden, The Netherlands;; § Department of Anatomy & Embryology, Leiden University Medical Center, Leiden, The Netherlands;; ‖ Neurosurgical Department, Neurological Center at the American British Cowdray Medical Center, Mexico City, Mexico;; ¶ Department of Neurosurgery, Alrijne Medical Center, Leiden, The Netherlands

**Keywords:** Hand anatomy, Hypothenar fat pad, Nerve of Henle, Open carpal tunnel release, Pacinian corpuscle, Palmar branch ulnar nerve, Ulnar artery

## Abstract

**BACKGROUND AND OBJECTIVES:**

Postoperative pain may occur following open carpal tunnel release (OCTR). Various causes have been postulated. During OCTR, adipose tissue located between the palmar aponeurosis and the flexor retinaculum is exposed. It is unknown whether damage to this pad of supraretinacular fat (SRF) might contribute to postoperative palmar pain or tenderness. We studied the sensory innervation of the SRF exposed in OCTR to assess whether SRF damage could potentially generate pain.

**METHODS:**

A microanatomic dissection of the innervation and vascular supply of the SRF was performed in 25 embalmed human cadaveric upper limbs. Eight fat pads were removed en bloc for histological evaluation. Three-dimensional reconstructions were made based on immunohistochemically stained sections using computer-assisted microscopy.

**RESULTS:**

The SRF is the radial continuation of the hypothenar fat pad that covers the neurovascular bundle in the Guyon canal. The fat pad is richly innervated and contains Pacinian corpuscles. The sensory innervation originates exclusively from the ulnar nerve (palmar branch) and its vascular supply from the ulnar artery. The integrity of the SRF can be preserved by detaching it from the flexor retinaculum in a radial to ulnar fashion.

**CONCLUSION:**

The SRF, which is exposed during OCTR, is richly innervated by sensory fibers from the ulnar nerve. It is the radialmost extension of the hypothenar fat pad. In view of its rich innervation, damage to the SRF during OCTR might generate postoperative pain. Preserving its integrity during OCTR is technically possible and even simplifies the procedure. Clinical trials are needed to corroborate whether preservation of the SRF during OCTR indeed makes a clinical difference in postoperative pain generation.

## Graphical abstract

**Figure:**
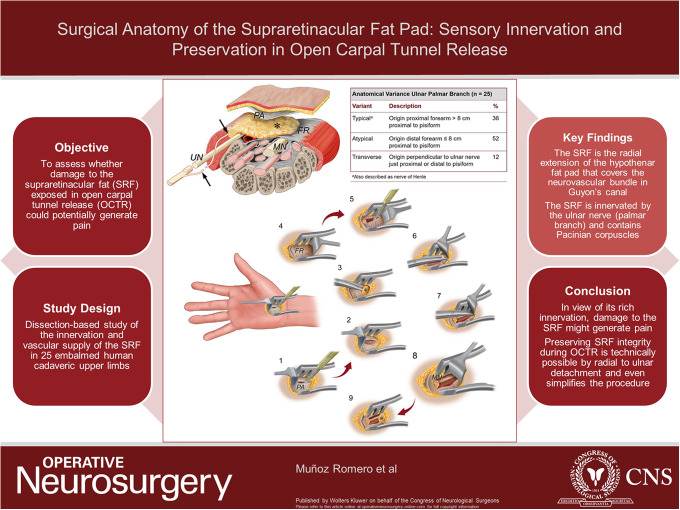

**ABBREVIATIONS:**
CTS: carpal tunnel syndrome
FR: flexor retinaculum
HE: hematoxylin-eosin
OCTR: open carpal tunnel release
PB: palmar branch
SRF: supraretinacular fat.

Carpal tunnel syndrome (CTS) is the most common nerve entrapment mononeuropathy with a prevalence of around 4%.^1^ Approximately 1 of 6 patients with CTS undergoes surgery for CTS during their lifetime.^2^ Median nerve decompression can be performed either open or endoscopically with comparable clinical outcomes.^3^ An unwanted postoperative side effect of surgery is persistent palmar pain of which 2 different types are distinguished: “pillar pain” and postoperative scar tenderness. “Pillar pain” is described as a deep-seated ache referred to the thenar or hypothenar eminences, which may occur independent of the applied technique.^4,5^ A clear definition of this painful phenomenon is lacking and the incidence is, therefore, difficult to assess.^6^ Theories concerning the etiology of pillar pain can be divided into 4 categories. These are (1) ligamentous or muscular, (2) structural alterations in the carpal arch, (3) neurogenic, and (4) edematous.^7^ Postoperative scar tenderness may be due to iatrogenic injury to small cutaneous palmar branches (PBs) of the ulnar and median nerves transected during incision (Figure 1).^5^ Anatomic dissections have demonstrated their origin from the ulnar and median PBs and from the superficial branch of the ulnar nerve.^8-11^ Preservation of these cutaneous branches in open carpal tunnel release (OCTR) has been claimed to reduce the incidence and severity of scar pain,^12,13^ but others found no difference.^14^

**FIGURE 1.**
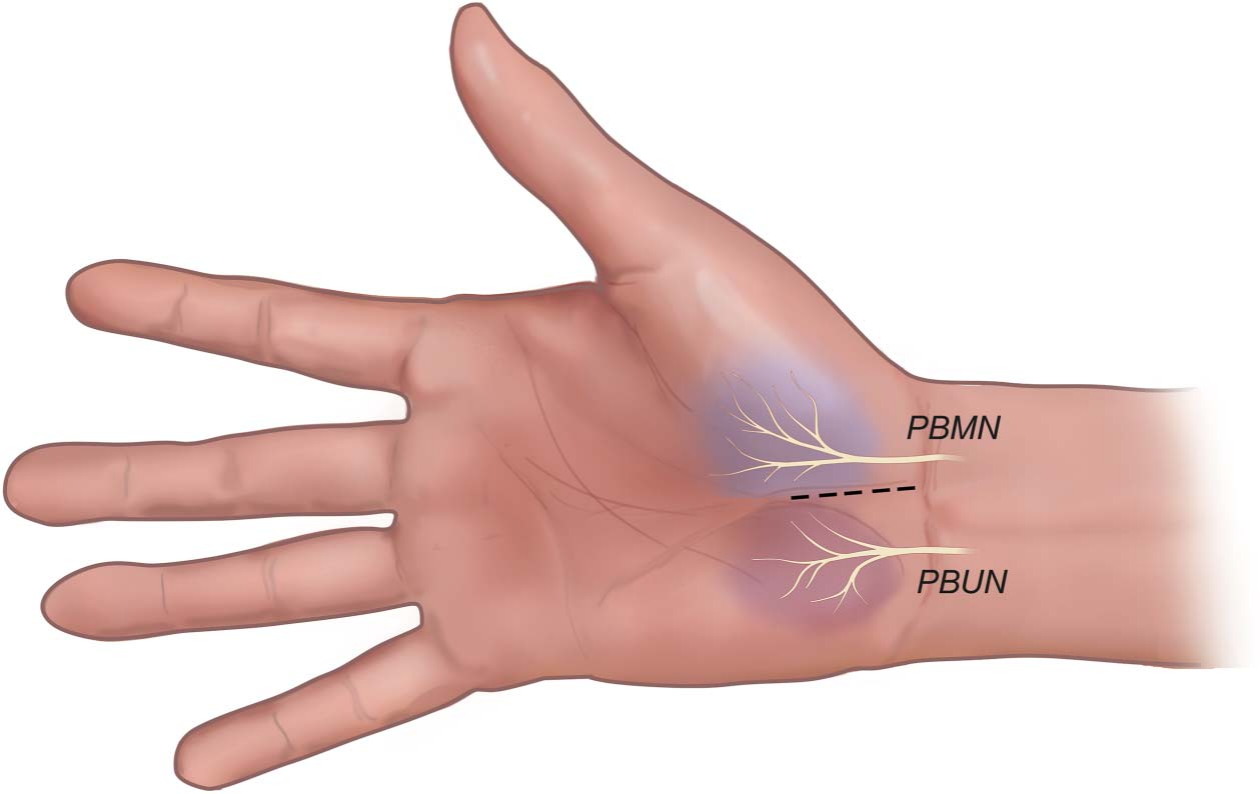
Artistic illustration showing the cutaneous innervation of the proximal palm by the PBs of the ulnar and median nerves (palmar aspect, right hand). The skin at the base of the hypothenar eminence (shaded dark red) and that of the thenar eminence (shaded blue) are supplied by terminal branches of the ulnar and median PBs, respectively. These cutaneous nerve branches may be at risk in open carpal tunnel release for transection during skin incision (dashed line), resulting in postoperative scar tenderness. PB, palmar branch; PBMN, palmar branch median nerve; PBUN, palmar branch ulnar nerve. *Copyright Martijn J. A. Malessy. Published with permission.*

During OCTR, adipose tissue located between the palmar aponeurosis and the flexor retinaculum (FR) is exposed (Figure 2). This supraretinacular fat (SRF) should not be confused with the palmar fat underneath the most distal fibers of the FR, covering the sensory digital branches of the median nerve.^15-17^

**FIGURE 2.**
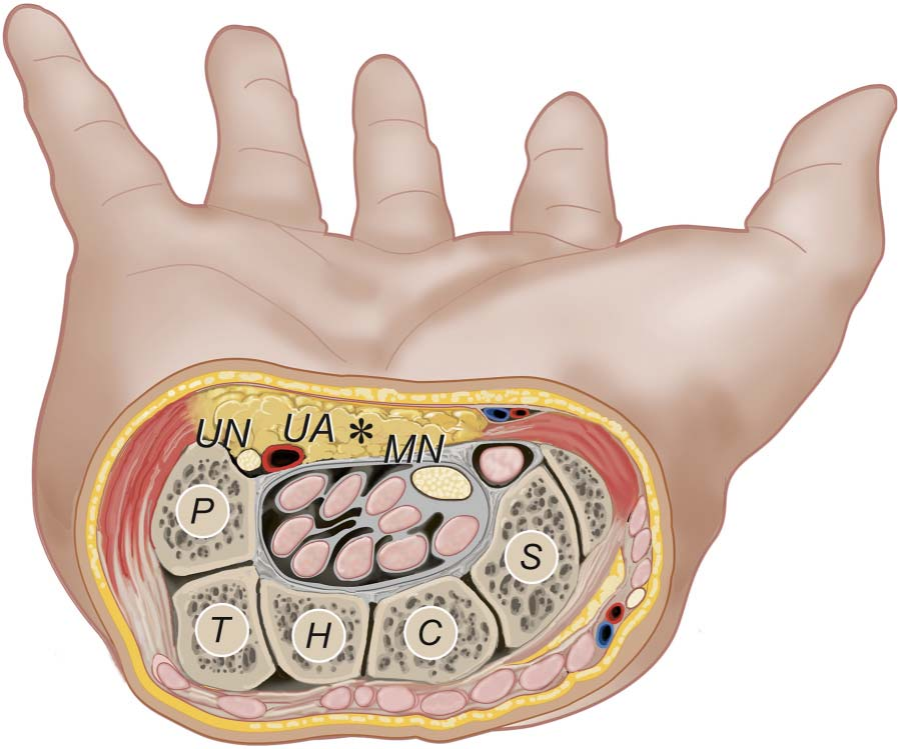
Artistic illustration showing the SRF exposed in open carpal tunnel release: cross-section of the wrist (right hand) at the level of the carpal tunnel (proximal view). The SRF is indicated by an asterisk. Carpal bones: C, capitate; H, hamate; P, pisiform; S, scaphoid; T, triquetrum; MN, median nerve; SRF, supraretinacular fat; UA, ulnar artery; UN, ulnar nerve. *Copyright Martijn J. A. Malessy. Published with permission.*

Whether damage to the SRF exposed in OCTR might contribute to postoperative palmar pain or tenderness is, to our knowledge, unknown. Surgical descriptions on how to handle this fat pad during OCTR are scarce. Either it is not mentioned,^14,18-25^ or it is not distinguished from the subcutaneous fat and dissected either bluntly^26^ or sharply,^17^ or it is released in a further unspecified way,^27^ or it is retracted through.^28^ We could only find 1 paper published in the French literature in which a modified OCTR approach is proposed that respects the integrity of the SRF so as to minimize the extent of scar tenderness.^29^ This paper did not receive wide attention. We performed a microanatomic study of the SRF exposed in OCTR with a specific focus on its neurovascular supply. We provide arguments to maintain the integrity of the SRF to avoid pain generation and describe the surgical technique to enable this.

## METHODS

### Anatomic Dissection

A microanatomic dissection of the carpal tunnel region was performed in 25 embalmed human cadaveric upper limbs (12 right and 13 left limbs from 11 male and 11 female Caucasian donors; mean age ± SD, 83.0 ± 9.2 years; range, 52-97 years) at the anatomic skills laboratory in our university medical center between October 2013 and April 2014 and March and June 2020. Body donor consent, including written informed consent for the publication of cadaveric images, was obtained from all donors. Embalmment was within 36 h after death by femoral artery perfusion with a 2% to 4% formaldehyde solution. Limbs were randomly selected, excluding upper limbs with externally visible abnormalities. In 4 upper limbs, the brachial artery was injected with red-dyed silicone (PlatSil® 73-15; Polytek Development Corp) after fixation. The authors state that every effort was made to follow all local and international ethical guidelines and laws that pertain to the use of human cadaveric donors in anatomic research.^30^ Institutional review board approval was not required for this study. The study adhered to the guidelines for reporting original anatomic studies (AQUA Checklist).^31^

**FIGURE 3.**
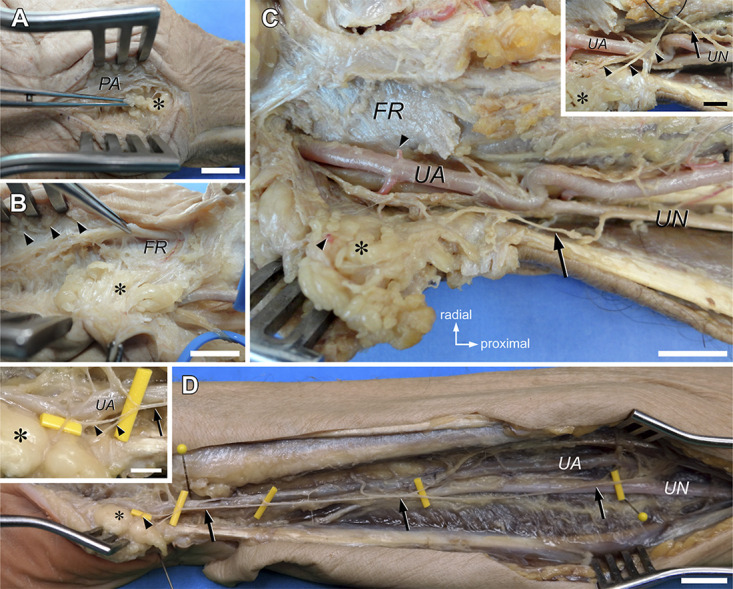
Microanatomic dissection of the supraretinacular fat exposed in open carpal tunnel release. **A** and **B**, Photographs of a cadaveric right upper limb at the level of the wrist (palmar view), showing the topography of the fat pad after opening of the palmar skin (orientation as indicated in panel **C)**. The fat pad (asterisk) is seen as a distinct structure located between the PA and FR and is distinguished from the subcutaneous fat (**B**, arrowheads) by its yellow color and lobulated appearance. Scale bars = 10 mm. **C** and **D**, Photographs of cadaveric left upper limbs at the level of the wrist and (distal) forearm (palmar views), showing the neurovascular supply of the hypothenar fat pad. The images are flipped left-right to facilitate comparison with panels **A** and **B**. The fat pad (asterisk) is dissected and reflected ulnarward, exposing the neurovascular bundle within the Guyon canal. The blood supply to the fat pad is from the UA (injected in **C** with red-dyed PlatSil® 73-15 silicone); a segmental branch (transected in **C**) to the fat pad is indicated by (an) arrowhead(s). The nerve supply is from the ulnar PB (arrows) (**C**, atypical variant; **D**, typical variant). The inset in **C**, **D** (same specimens; ulnar PB retracted in **C**) shows the PB (arrow) supplying several smaller branches (arrowheads) to the base of the fat pad (asterisk). Scale bar = 10 mm; scale bar (insets) = 5 mm. FR, flexor retinaculum; PA, palmar aponeurosis; PB, palmar branch; UA, ulnar artery; UN, ulnar nerve.

Dissections were performed with microsurgical instruments under ×2.5 loupe magnification or using a Zeiss Universal S2 operating microscope (Carl Zeiss AG) at ×10 to ×40 magnification. All dissections were collectively made by an experienced neurosurgeon (first or last author) and a neuroanatomist. A standard OCTR approach^15^ was used with a longitudinal skin incision of 4 cm (roughly twice the intraoperative length) to open the palm. The subcutaneous fat and palmar aponeurosis were carefully freed. The SRF below the palmar aponeurosis was distinguished from the subcutaneous fat by its intense yellow color (cf, Ling et al^17^) and its lobulated and smooth aspect (Figure 3A and 3B).

The skin of the forearm was opened with a longitudinal incision starting at the cubital fossa and extending to the distal wrist crease. The ulnar nerve and ulnar artery were identified along the flexor carpi ulnaris and dissected from the cubital tunnel to the Guyon canal. The median nerve and its PB were identified in the distal third of the forearm between the tendons of flexor pollicis longus and flexor digitorum superficialis and dissected to the carpal tunnel. The radial artery was identified and dissected to the thenar area.

The SRF was carefully dissected. Great care was taken to identify, preserve, and follow small nerve branches and vessels to the SRF. The origin of nerve branches was evaluated relating to those nerves known to provide sensory innervation to the carpal tunnel region: PB of ulnar nerve,^9,10,32^ nerve of Henle,^10,33,34^ “transverse” PB of ulnar nerve,^10^ and PB of median nerve.^8,11^ Ulnar nerve branches were classified following Martin et al^10^ and McCabe and Kleinert^33^ (Table). The point of origin of ulnar nerve branches was measured relative to the center of the pisiform bone with the forearm in supination. Measurements were made with a ruler.

**TABLE.** Origin and Classification of Ulnar Nerve Branches Innervating the Supraretinacular Fat Exposed in Open Carpal Tunnel Release

Nerve branch^a^	Origin relative to pisiform (cm)		n	%
	Mean ± SD	Range		
Ulnar PB—typical variant^b^	15.71 ± 3.05	10.0-20.0	9	36
Ulnar PB—atypical variant	3.63 ± 1.72	1.5-6.8	13	52
Transverse ulnar PB	−0.33 ± 0.38	−0.6-0.1	3	12

PB, palmar branch.

a Ulnar PBs were classified following Martin et al^10^ and McCabe and Kleinert^33^ and typed as typical if they exited the ulnar nerve in the proximal forearm more than 8 cm proximal to the center of the pisiform bone, as atypical if they exited the ulnar nerve in the distal 8 cm of the forearm relative to the pisiform, or as “transverse” if they exited the ulnar nerve perpendicular to its longitudinal axis just proximal to or distal to (shown as the negative value) the pisiform.

b Also described as the nerve of Henle.^10,33,34^

### Nomenclature

As pointed out by others,^32,33,35^ nerve of Henle and PB of ulnar nerve may well be descriptors of the same branch of the ulnar nerve, which is present either as a typical (proximal) or atypical (distal) branch. Although not included in the *Terminologia Anatomica*,^36^ here we refer to the nerve of Henle as the eponym of PB of ulnar nerve.

### Histology and Immunohistochemistry

In 8 specimens (4 males: 2 left and 2 right limbs; 4 females: 2 left and 2 right limbs), the SRF was removed en bloc with its innervation (4 typical, 4 atypical ulnar PBs) and vascular supply, stored in 70% ethanol at 4°C, embedded in paraffin using standard procedures for formalin-fixed tissues, and cut into 7-µm-thick transverse serial sections. Tissue sections were dewaxed and either stained with hematoxylin-eosin (HE) or immunostained with a monoclonal mouse antineurofilament protein antibody (1:500; clone 2F11; M0762; Dako Denmark A/S), a polyclonal rabbit anti-S100 serum (1:8000; Z0311; Dako Denmark A/S) or a monoclonal mouse anti-α smooth muscle actin antibody (1:5000; clone 1A4; A2547; Sigma-Aldrich). Immunostaining was performed as described,^37^ except that for staining with the anti-α smooth muscle actin antibody, a two-step indirect method was used. The second layer antisera included biotinylated rabbit antimouse (1:200; E0354; Dako Denmark A/S) and biotinylated goat antirabbit (1:200; BA-1000; Vector Laboratories, Inc) IgG serum, and peroxidase-conjugated rabbit antimouse IgG serum (1:250; P0260; Dako Denmark A/S).

### Three-Dimensional Reconstructions

Computer-assisted 3-dimensional reconstructions were prepared and displayed in Amira® imaging software 5.3.3 (Template Graphics Software; Visage Imaging) using a section interval of 70 µm. Digital images of serial sections were obtained by whole-slide imaging using a Philips Ultra-Fast Scanner 1.6 RA and Philips Image Management System 2.3.1.1 (Philips Digital Pathology Solutions).

## RESULTS

### Macroscopic Anatomy

The SRF is the radial continuation of the hypothenar fat pad that covers the neurovascular bundle in the Guyon canal (Figures 2 and 3). The fat pad extends radially in varying degrees from under the palmar carpal ligament, while roofing the central segment of the Guyon canal.^38^ It is loosely attached to the FR under the palmar aponeurosis fibers.

The blood supply of the fat pad originated solely from the ulnar artery and consisted of up to 4 segmental branches (Figure 3C and 3D). In all specimens, the innervation came from the ulnar nerve, either as a typical (9/25; 36%) (Figure 3D) or atypical (13/25; 52%) (Figure 3C) PB or as a “transverse” PB (3/25; 12%) (Table). In 1 specimen with an atypical ulnar PB, the fat pad received an additional transverse branch from the ulnar nerve just distal to the pisiform within the Guyon canal. The PB gave off numerous small branches to the base of the fat pad at the level of the pisiform bone (Figure 3C and 3D; inset). In none of the specimens did we identify a contribution to the innervation of the fat pad from the median nerve or from its PB.

### Microscopic Anatomy

**FIGURE 4.**
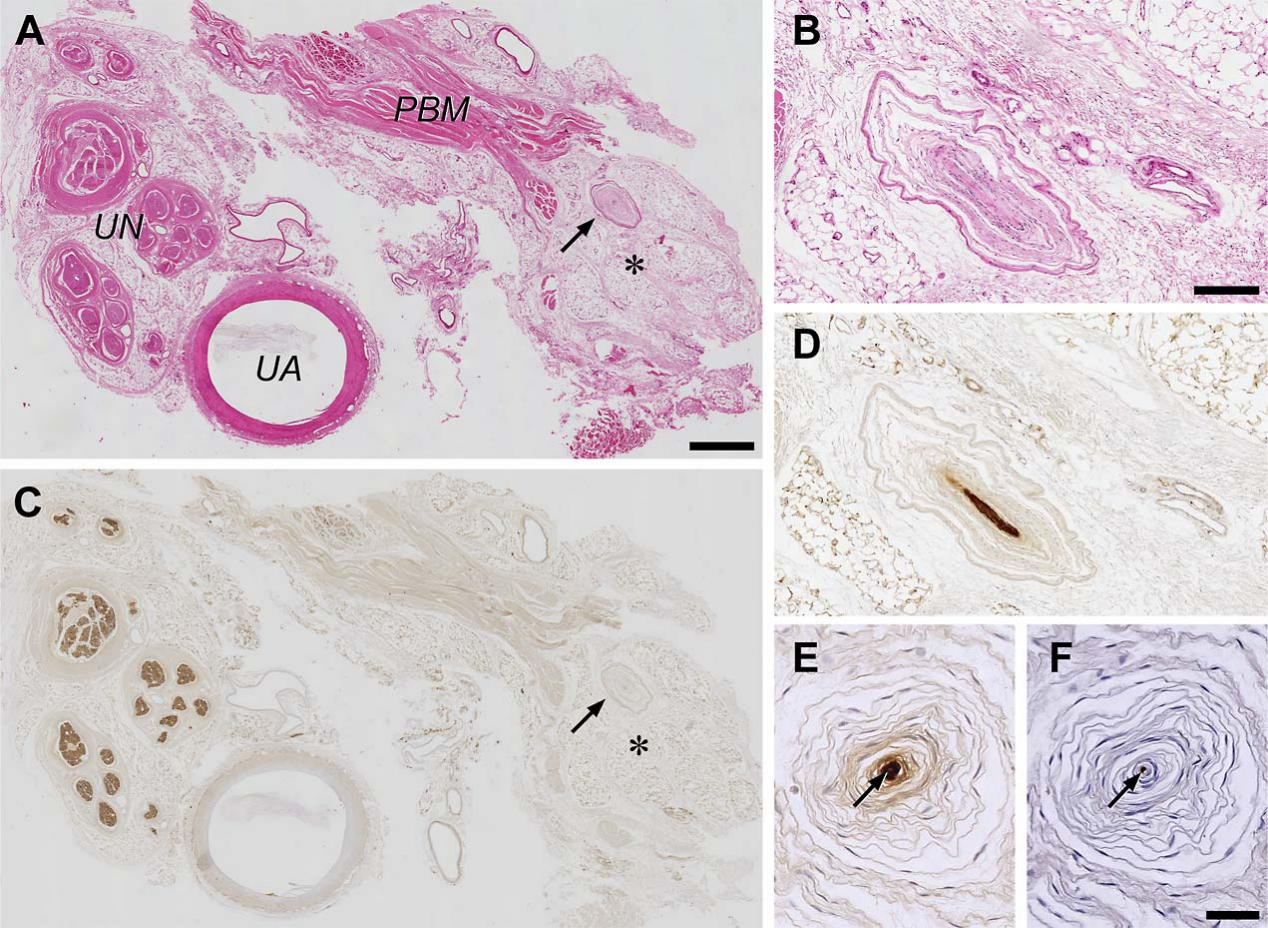
Histology of the supraretinacular fat exposed in open carpal tunnel release. Photomicrographs of serial tissue sections containing the fat pad, UA, and UN from a cadaveric right upper limb stained with hematoxylin-eosin **A** and **B**, the anti-S100 protein antibody (staining of Schwann cells) **C**-**E**, and the antineurofilament protein antibody (staining of axons) **F**. **A** and **C**, Low-power photomicrographs of adjacent tissue sections, showing a Pacinian corpuscle (arrow) within the fat pad (asterisk) some distance from muscle fiber bundles of the PBM. Scale bar = 1 mm. **B**, **D**-**F**, High-power photomicrographs of Pacinian corpuscles located within the fat pad, showing the typical structure of a concentric multilayered outer core and capsule **B** around a S100 protein-positive inner core of periaxonic lamellar cells **D**, **E**, and containing a neurofilament protein-positive central nerve fiber **F**. Panels **B** and **D**, adjacent oblique sections of a Pacinian corpuscle. Scale bar = 250 µm. Panels **E** and **F**, adjacent cross-sections of a Pacinian corpuscle. Arrow, central nerve fiber. Scale bar = 50 µm. PBM, palmaris brevis muscle; UA, ulnar artery; UN, ulnar nerve.

**FIGURE 5.**
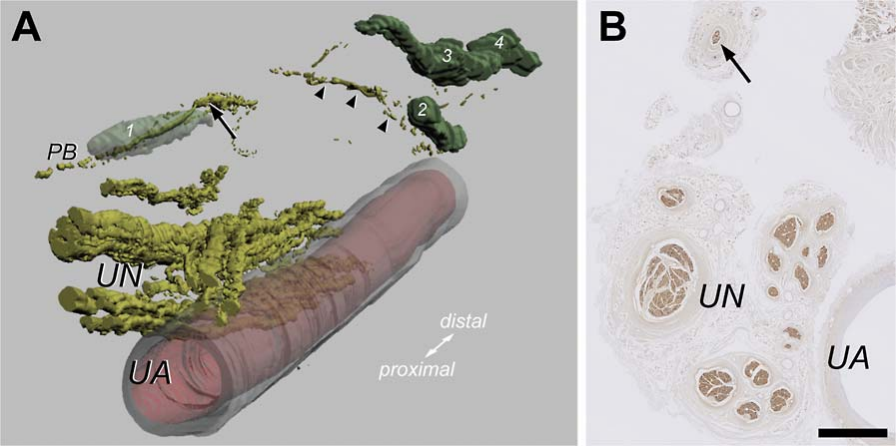
Innervation of the supraretinacular fat exposed in open carpal tunnel release. **A**, Three-dimensional reconstruction of immuno- and HE-stained tissue sections containing the hypothenar fat pad, UA, and UN from a cadaveric right upper limb. Three entities were reconstructed, viz, (1) the vessel wall of the UA in transparent red (anti-α smooth muscle actin antibody), (2) the contours of the UN (fascicles) and its PB in yellow (anti-S100 protein antibody), and (3) the contours of 4 Pacinian corpuscles (labeled 1-4) in (transparent) green (hematoxylin-eosin). Each reconstruction was the result of 141, 7-µm-thick sections, using an interval of 70 µm between consecutive sections. **B**, Photomicrograph of an anti-S100 protein-stained tissue section used for the reconstruction. The arrow in **A** and **B** indicates the ulnar PB (atypical variant) at a level common to both panels. Pacinian corpuscles within the fat pad are innervated by terminal branches from the ulnar PB (arrowheads). The reconstructed Pacinian corpuscles (n = 4) measured 2.05 ± 0.69 mm (mean length ± SD; range, 1.19 to 2.87 mm) × 1.08 ± 0.18 mm (mean width ± SD; range, 0.86 to 1.24 mm) in diameter. Scale bar = 1 mm. PB, palmar branch; UA, ulnar artery; UN, ulnar nerve.

Examination of HE-stained tissue sections revealed the presence of Pacinian corpuscles within the fat pad, measuring up to 1 to 3 mm in length, and distributed singly or in small clusters (Figures 4 and 5). Most Pacinian corpuscles within the fat pad were found at the level of the Guyon canal, either superficial near proximal muscle fiber bundles of palmaris brevis bordering the central segment of the roof of the Guyon canal distally (Figure 4A and 4C) (cf, Bramke and May^39^) or deep near the ulnar artery (Figure 5A) (cf, Roset-Llobet and Domenech-Mateu^40^). The central nerve fiber and accompanying Schwann sheath comprising the inner core of the corpuscles could readily be visualized by their immunoreactivity for neurofilament protein (Figure 4F) and S100 protein (Figure 4D and 4E), respectively.^41,42^ Three-dimensional reconstruction of S100 protein-stained sections showed that the central nerve fiber of the corpuscles originated from the PB of the ulnar nerve (Figure 5). No other mechanosensory end organs (eg, Merkel cells, Meissner corpuscles, or Ruffini endings) were found within the fat pad.

## DISCUSSION

### Key Findings

**FIGURE 6.**
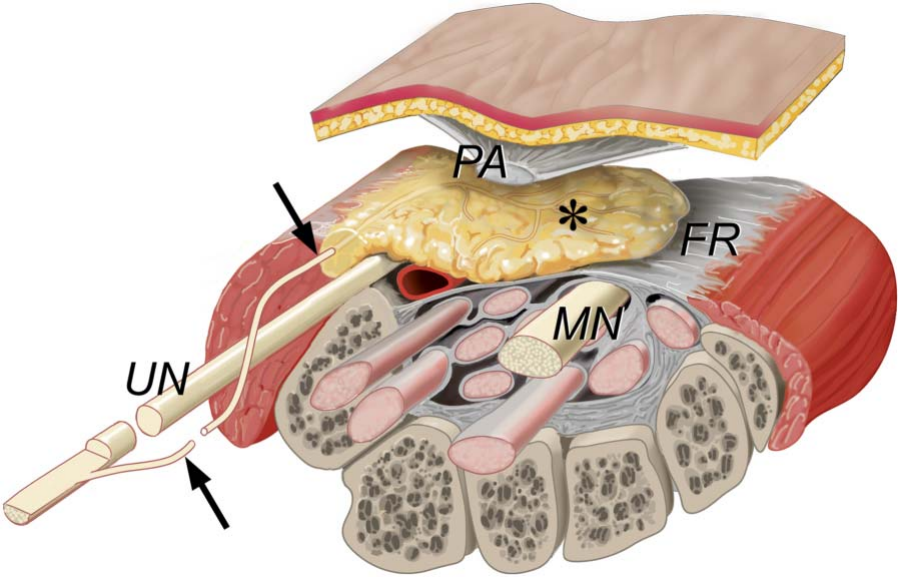
Artistic illustration reflecting the findings of the anatomic dissections and histologically demonstrated innervation of the SRF exposed in open carpal tunnel release—carpal tunnel (right hand): structure and anatomic relations. The SRF (asterisk) between the PA and the FR is the radial continuation of the hypothenar fat pad that covers the neurovascular bundle in the Guyon canal. It is innervated by the ulnar PB (arrows), either as a typical (proximal) or atypical (distal) nerve, or as a “transverse” PB (not shown). FR, flexor retinaculum; MN, median nerve; PA, palmar aponeurosis; PB, palmar branch; SRF, supraretinacular fat; UN, ulnar nerve. *Copyright Martijn J. A. Malessy. Published with permission.*

The SRF, which is exposed during OCTR, is the radial continuation of the hypothenar fat pad that covers the neurovascular bundle in the Guyon canal.^38^ The sensory innervation exclusively originates from the ulnar nerve (PB) and its segmental blood supply from the ulnar artery (Figure 6). The fat pad is richly innervated and contains Pacinian corpuscles. The radial part of the fat pad is loosely attached to the FR.

### Interpretation

**FIGURE 7.**
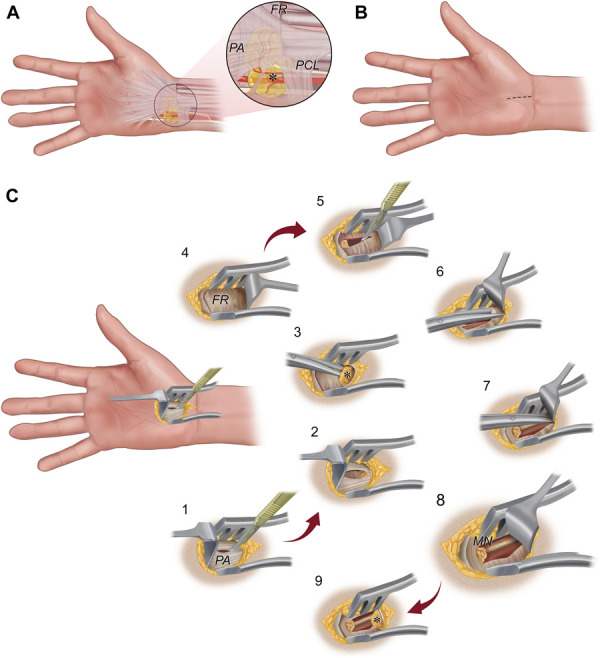
Artistic illustrations demonstrating the authors' surgical technique to expose and preserve the SRF during OCTR. **A**, Fat pad overlying the FR under the PA is the radial continuation of the hypothenar fat pad under the distal extension of the antebrachial fascia (PCL) and roofing the central segment of the Guyon canal (asterisk). It is loosely attached to the FR under the PA fibers and extends in varying degrees from ulnar to radial. **B**, Short palmar skin incision (2-3 cm) (dashed line) for OCTR is made just ulnar of the thenar crease in line with the longitudinal axis of the ring finger, beginning at the intersection of Kaplan line and ending just distal to the distal wrist crease.^9,15^ **C**, Surgical technique: **C1**, subcutaneous fat is bluntly dissected to preserve cutaneous palmar branches of the ulnar and median nerves. The subcutaneous fat is retracted with a blade retractor, and the PA is split along its fiber direction by sharp dissection. **C2**, SRF below the PA is exposed proximally. **C3**, PA is retracted. The SRF (asterisk) is further exposed, detached from the FR at its radial border, and mobilized ulnarward. **C4**, FR is exposed by retracting the mobilized fat pad with a blade retractor in a proximal-ulnar direction. The integrity and neurovascular supply of the SRF is thereby left intact. **C5**, FR is first sectioned at its ulnar distal aspect. Fatty tissue appears from underneath the FR (“fat pad” sign) when the distal FR is released.^15,16^ **C6**, **C7**, Proximal aspect of the FR is further exposed by retracting the fat pad bluntly in a proximal, upward and ulnar direction. The proximal part of the FR is opened with scissors with its tips ulnar of the median nerve. **C8**, To completely decompress the MN in the carpal tunnel, the proximal edge of the FR is cut under direct vision and is released entirely if the distal antebrachial fascia is reached, which is 2 to 3 cm proximal to the skin incision. **C9**, Blade retractor is removed, and the SRF (asterisk) returns to its original position. FR, flexor retinaculum; MN, median nerve; OCTR, open carpal tunnel release; PA, palmar aponeurosis; PCL, palmar carpal ligament; SRF, supraretinacular fat. *Copyright Martijn J. A. Malessy. Published with permission.*

The hypothenar fat pad is a discrete anatomic structure with a proposed protective function to the ulnar neurovascular bundle at the wrist when spherical-shaped objects are firmly compressed into the palm.^43^ In view of its sensory innervation with Pacinian corpuscle receptors, the fat pad may also have a role during spherical grip in the perception of vibratory pressure.^44^ Preservation during OCTR, hypothenar fat pad transposition,^45,46^ or any type of hand surgery seems relevant not only to avoid function loss but also to prevent palmar discomfort or pain generation. During OCTR, the radial part of the fat pad can be easily mobilized from radial to ulnar. In doing so, the integrity of the SRF and its vascularization and innervation, which is from the ulnar side, remain intact (Figure 7). Keeping the fat pad intact may be relevant not only for OCTR but also for endoscopic approaches.^47,48^

The PB of the ulnar nerve (or nerve of Henle) is known for its sympathetic innervation to the ulnar artery^34^ and sensory cutaneous innervation to the proximal medial palm.^9,10,32-34^ Our study is, to our knowledge, the first to describe the ulnar PB to additionally innervate Pacinian corpuscles. The corpuscles within the fat pad had a normal morphology^49^ and were related to terminal branches of the ulnar PB using 3-dimensional reconstruction of immunostained sections. Other types of sensory receptors were not observed in the fat pad.

Pacinian corpuscle afferents act as low-threshold mechanoreceptors sensitive to high-frequency stimulation and usually do not respond to painful stimuli.^44^ Normally, an Aβ-fiber is present at the center of the corpuscle, and pain is transmitted through nociceptive C- and Aδ-fibers. Following damage to the SRF during OCTR, however, Pacinian corpuscle hyperplasia may develop due to trauma.^50^ Pacinian hyperplasia is painful, and it has been shown that the Aβ-fibers then express calcitonin gene-related peptide and substance P.^51^ Both neuropeptides are involved in pain perception. This mechanism, although unproven like all other explanations for palmar pain and discomfort following OCTR, is an addition to pillar pain and postoperative scar tenderness^4,5^ or might even be a part of the former, as suggested by Wilson.^52^

### Implications

During revision surgery for recurrent CTS, the hypothenar fat pad is sometimes used as an adjunct to neurolysis to prevent readherence and perineural scarring of the median nerve. Once mobilized, the fat pad flap is interposed between the median nerve and the radial wall of the carpal tunnel and secured deep to the radial leaf of the FR.^45,46^ Although hypothenar fat pad transposition may yield clinically relevant improvement for recurrent CTS as compared with other surgical options,^53,54^ its interoperative indications should be considered critically.^55^ In view of our findings, transposing the fat pad risks stretching or damaging its neural supply and associated Pacinian corpuscles, which might induce palmar pain or compromise the function of the latter as a pressor sensor. In addition, mechanosensory feedback during gripping might diminish when the fad pad is secured deep to the carpal tunnel.

### Limitations

Only a randomized controlled trial can address whether surgical preservation of the SRF contributes to a decrease in postoperative palmar pain incidence. OCTR with careful handling and preservation of the hypothenar fat pad (Figure 7) should then be compared with traditional OCTR with either sharp or blunt dissection of the SRF below the palmar aponeurosis to expose the FR. Similar clinical trials are being conducted for other fat pads to optimize surgical strategy and to improve postoperative outcomes.^56^ Although it seems difficult to advocate a different approach to a common surgical procedure without any clinical evidence, we have noticed 2 surgical technical advantages of mobilizing the SRF from radial to ulnar (Figure 7). SRF detachment and keeping it out of the way allows for a clear visualization of the entire FR, while preserving meticulous hemostasis. Thereby, the completeness of the FR release and median nerve decompression is far better to assess and, in fact, these technical nuances make OCTR an even easier procedure. Moreover, after the FR is sectioned and the retractor blade is removed, the SRF falls back on top of the exposed surface of the median nerve and the sectioned ulnar and radial edges of the divided FR. Thereby, nerve coverage and vascularization of the area are secured. One of the causes of recurrent symptoms following OCTR is epineural fibrous fixation of the median nerve, secondary to the formation of postoperative adhesions or cicatricial tethering.^57^ Whether careful handling of the SRF during OCTR reduces the occurrence of recurrent median nerve symptoms by diminishing postoperative scar formation and retaining free nerve sliding has not been studied.

Our results correspond to those of Roure and Masquelet.^29^ They also found an exclusive ulnar-based neurovascular supply of the hypothenar fat pad but did not confirm their findings with histology. In their study, the innervation was predominantly from branches originating from the ulnar nerve within the Guyon canal arising either directly from the ulnar nerve proper or from its superficial branch. Both this study and their study comprise only a limited sample size. Therefore, a contribution of the median nerve to the innervation of the fat pad, albeit with a low frequency, cannot be completely excluded. The PB of the median nerve may have a more ulnar distribution if a (typical) ulnar PB is lacking.^32^ Whether in such cases the hypothenar fat pad has a dual innervation awaits further study.

## CONCLUSION

During OCTR, the radial extension of the hypothenar fat pad is exposed. The innervation and vascularization of the fat pad is from the ulnar nerve and ulnar artery, respectively. In view of its innervation with Pacinian corpuscle receptors, keeping the fat pad intact is likely important. Because the radial part of the fat pad is loosely attached to the FR, its integrity can be preserved by radial to ulnar detachment. This might not only reduce postoperative pain but also facilitate exposure of the FR, thus simplifying OCTR. Clinical trials are needed to corroborate whether preservation of the SRF during OCTR indeed makes a clinical difference in postoperative pain generation.

## COMMENTS

The authors delineate the anatomic nuances and the clinical significance of the supraretinacular fat pad during open carpal tunnel release (OCTR). This research provides an anatomic and functional rationale for preserving this often-overlooked structure to potentially mitigate postoperative palmar pain, a common yet challenging complication of OCTR.

Utilizing a rigorous dissection methodology on embalmed cadaveric specimens, the study brings to light the detailed sensory innervation and vascular supply of the fat pad, findings that are important for understanding its role in postoperative outcomes. The authors' observations that the fat pad is primarily innervated by the ulnar nerve and receives its blood supply from the ulnar artery are not only of anatomic interest but also have clinical implications. By highlighting the potential link between the structural integrity of this fat pad and the reduction of postoperative pain, the study sets a compelling argument for the modification of current surgical techniques in OCTR. Additionally, the reduced invasiveness of endoscopic and percutaneous methods might offer advantages in terms of preserving this fat pad and potentially reducing postoperative pain. Moreover, these findings pave the way for future clinical research. The authors astutely call for clinical trials to validate whether the preservation of the supraretinacular fat pad during OCTR can indeed translate into better clinical outcomes, a suggestion that is both timely and scientifically sound.

The incorporation of high-resolution imaging and histological analysis adds a significant depth to the manuscript, offering clear and instructive insights into the microanatomy of the region. Such detailed visualization aids in the educational dissemination of complex anatomic relationships, which are crucial for both trainees and practicing surgeons aiming to refine their surgical techniques.

Furthermore, the study raises intriguing possibilities for analogous anatomic studies in other regions, such as the scalene fat pad during brachial plexus exposure; by understanding its neural and vascular anatomy, surgeons might develop strategies that enhance mobilization while minimizing trauma, thereby improving surgical outcomes.

**Ken M. Porche and Robert J. Spinner**

*Rochester, Minnesota, USA*
